# Graphene-Oxide-Based Electrochemical Sensors for the Sensitive Detection of Pharmaceutical Drug Naproxen

**DOI:** 10.3390/s20051252

**Published:** 2020-02-25

**Authors:** Lanting Qian, Antony Raj Thiruppathi, Reem Elmahdy, Joshua van der Zalm, Aicheng Chen

**Affiliations:** Electrochemical Technology Centre, Department of Chemistry, University of Guelph, 50 Stone Road East, Guelph, ON N1G 2W1, Canada; qianl@uoguelph.ca (L.Q.); athirupp@uoguelph.ca (A.R.T.); relmahdy@uoguelph.ca (R.E.); vanderzj@uoguelph.ca (J.v.d.Z.)

**Keywords:** naproxen, pharmaceutical drug, differential pulse voltammetry, electrochemical sensor, graphene oxide, doping

## Abstract

Here we report on a selective and sensitive graphene-oxide-based electrochemical sensor for the detection of naproxen. The effects of doping and oxygen content of various graphene oxide (GO)-based nanomaterials on their respective electrochemical behaviors were investigated and rationalized. The synthesized GO and GO-based nanomaterials were characterized using a field-emission scanning electron microscope, while the associated amounts of the dopant heteroatoms and oxygen were quantified using x-ray photoelectron spectroscopy. The electrochemical behaviors of the GO, fluorine-doped graphene oxide (F-GO), boron-doped partially reduced graphene oxide (B-rGO), nitrogen-doped partially reduced graphene oxide (N-rGO), and thermally reduced graphene oxide (TrGO) were studied and compared via cyclic voltammetry (CV) and differential pulse voltammetry (DPV). It was found that GO exhibited the highest signal for the electrochemical detection of naproxen when compared with the other GO-based nanomaterials explored in the present study. This was primarily due to the presence of the additional oxygen content in the GO, which facilitated the catalytic oxidation of naproxen. The GO-based electrochemical sensor exhibited a wide linear range (10 µM–1 mM), a high sensitivity (0.60 µAµM^−1^cm^−2^), high selectivity and a strong anti-interference capacity over potential interfering species that may exist in a biological system for the detection of naproxen. In addition, the proposed GO-based electrochemical sensor was tested using actual pharmaceutical naproxen tablets without pretreatments, further demonstrating excellent sensitivity and selectivity. Moreover, this study provided insights into the participatory catalytic roles of the oxygen functional groups of the GO-based nanomaterials toward the electrochemical oxidation and sensing of naproxen.

## 1. Introduction

Naproxen (2-(6-methoxynaphthalen-2-yl) propanoic acid (S/R)) is a nonsteroidal anti-inflammatory drug (NSAID) that is used to treat inflammation, fever, rheumatoid arthritis, and stiffness. Naproxen inhibits COX-1 and COX-2 enzymes, which results in the inhibition of the synthesis of certain prostaglandins [1,2]. However, there are two major concerns associated with the use of naproxen. First, overuse can cause adverse side effects such as stomach pain, ulcers, and stomach bleeding [2]. Naproxen overdose may be initiated when an individual takes more than the recommended daily dosage. For instance, the recommended daily dosage of naproxen for temporary pain management using an oral immediate release tablet is 550 mg, followed by 275 mg orally every 6 to 8 hours, or 550 mg every 12 hours as needed, which should not exceed 1375 mg/day [3,4,5]. Cases of naproxen overdose include serious toxicity with seizures, altered cognitive status, and metabolic acidosis. Secondly, naproxen has recently been classified as an emerging pollutant in wastewater, in that significant concentrations have been found in the plasma and bile of fish exposed to treated effluent discharged by wastewater treatment plants [6]. Therefore, an economically viable, accurate, and rapid response sensor is urgently required for the protection of human/animal health and ecosystems.

Methods such as high-performance liquid chromatography (HPLC), UV-spectrophotometry, spectrofluorimetry, and mass spectrometry have been commonly employed to analyze pharmaceutical drugs; however, these techniques are generally time-consuming and costly. The electrochemical approach provides an accurate, fast, and cost-effective alternative to those detection methods [7]. For instance, Rossi et al. reported a cytochrome P450-based biosensor for the continuous monitoring of naproxen in real-time delivery system [8]. Afkhami et al. reported an enantioselective naproxen biosensor based on a chiral modified gold electrode decorated with gold nanoparticles [9]. Hendawy et al. reported nanomaterial-based carbon paste electrodes for the detection of naproxen and its degradation product [10]. Over the last decade, the exploration of graphene and graphene-based nanomaterials has garnered tremendous attention due to their unique electronic, structural, and physical attributes [11,12,13,14]. The structure of graphene includes a single layer of sp2-hybridized hexagonal lattices that form honeycomb structures with a high surface area, which are both electronically and thermally conductive [9]. Graphene oxide (GO) synthesized from graphite contains abundant oxygen functional groups, including hydroxyl, carboxylic acids, and epoxy groups, which demonstrate good catalytic activities, particularly for the oxidation of organic molecules [12,13,14,15]. However, the presence of oxygen groups disrupts the sp2 bonds of carbon rings, thereby lowering conductivity. This issue may be resolved by reducing graphene electrochemically, thermally, or chemically to generate reduced graphene oxide (rGO). This form of graphene exhibits a strong pi-electronic structure and higher conductivity, due to the cleavage of hydroxyl and epoxy functional groups, while retaining most of the carbonyl or carboxylic acid groups at its edges [16,17,18,19].

The reduction of GO can cause significant structural defects, which may play important roles in catalysis [19,20]. However, to the best of our knowledge, very few studies on how the oxygen functional groups and the defects affect the catalytic activity of GO and rGO have been reported. Therefore, it is vital to understand the catalytic attributes and kinetics that may be achieved by the oxygen groups of GO and rGO. These data can be then applied to design and tune the specific catalytic activity of GO-based nanomaterials for sensing and environmental applications [21,22,23]. In addition, over the last decade, heteroatom-doped GO materials have been extensively studied to tune the structural and electronic properties of GO for applications in energy storage and catalytic sensing [24,25,26,27].

The purpose of this study was three-fold: (i) to develop an advanced electrochemical sensor for the detection of naproxen; (ii) to compare the performance of various GO-based sensors and rationalize the effects of oxygenated functional groups; and (iii) to investigate the effects of dopants on the performance of the GO-based electrochemical sensors.

## 2. Materials and Methods

### 2.1. Reagents and Synthesis

Naproxen, glutamic acid, potassium nitrate, D-glucose, citric acid, sulfate ions, ascorbic acid and other reagents were purchased from Sigma-Aldrich. All experiments were conducted in a phosphate buffer solution (PBS) electrolyte. The PBS buffer was a mixture of pure water, Na_2_HPO_4_ and NaH_2_PO_4_, and NaOH was employed to adjust its pH to 7.2. GO was synthesized from high-purity graphite provided by ZEN Graphene Solutions Ltd., Thunder Bay, Canada, using a modified Hummers method [28]. GO was treated at 200 °C for 15 min in an oil bath to prepare thermally-reduced graphene oxide (TrGO). Fluorine-doped partially reduced graphene oxide (F-GO) was synthesized using a one-pot method reported by our group [29]. Boron-doped partially reduced graphene oxide (B-rGO) was synthesized from GO using a facile microwave method. Briefly, GO and boric acid were mixed at a 1:1 weight ratio first; the mixture was then subjected to microwave irradiation (1200W and NNST775S) for 2 min. The obtained B-rGO was washed with water and ethanol and dried at 50 °C overnight. Nitrogen-doped partially reduced graphene oxide (N-rGO) was synthesized using a hydrothermal method from GO and urea [30]. Briefly, GO was dispersed in water at 4.0 mg/ml by ultrasonication. Urea was added slowly while the GO dispersion was stirred. After being stirred for 1 h, the mixture was transferred to autoclave and subjected to hydrothermal treatment at 160 °C for 5 h. The obtained N-rGO was then washed with pure water and ethanol and finally dried at 50 °C overnight.

### 2.2. Electrode Preparation and Modification

The glassy carbon electrode (GCE) used in the experiments was polished using an alumina powder/water slurry, sonicated in acetone for one minute, and then in deionized water for three minutes, followed by water exchange and further sonication in deionized water for one minute. Subsequent to polishing and sonication, the electrode was characterized using potassium ferrocyanide (conc. 5 mM) in 0.2 M KNO_3_ using cyclic voltammetry (CV) in the potential range from −0.1 to 0.6 V to ensure that all electrodes were in pristine condition. Each of the GCE was tested and confirmed to be of a similar quality prior to modification. A 2.5-mg mass of GO, TrGO, B-rGO, F-GO, and N-rGO powders was dissolved in 1.0 ml pure water; 5.0 µL of the mixture was drop-cast onto the surface of the GCE and air dried for 3 h to obtain the modified electrodes. The GCE had a diameter of ~3.0 mm, with a surface area of 0.07 cm^2^.

### 2.3. Instrumentation and Methodology

The morphology and compositions of the fabricated GO, TrGO, F-GO, B-rGO, and N-rGO were characterized using a Hitachi SU-70 Schottky field emission scanning electron microscope (FE-SEM) and X-ray photoelectron spectroscopy (XPS) (Scienta Omicron Inc., Edmonton, Canada), respectively. CV and differential pulse voltammetry (DPV) were performed using a potentiostat (CHI-660D, CHI, USA). All electrochemical experiments were conducted using a three-electrode cell, where the GCE and the modified GCEs were employed as the working electrode. The auxiliary electrode was a platinum wire, whereas the reference electrode was a standard Ag/AgCl electrode (3 M KCl saturated with AgCl). A stock naproxen solution (conc. 20 mM) was prepared in 0.1 M NaOH to ensure complete dissolution of naproxen. All analytical quantifications were performed in a phosphate buffer solution (pH 7.2) at room temperature (22 ± 2 °C). All of the solutions were purged with pure argon gas (99.999%) for 20 minutes prior to the electrochemical measurements to remove any dissolved oxygen. 

## 3. Results and Discussion

### 3.1. Surface Characterization

**Figure 1:**
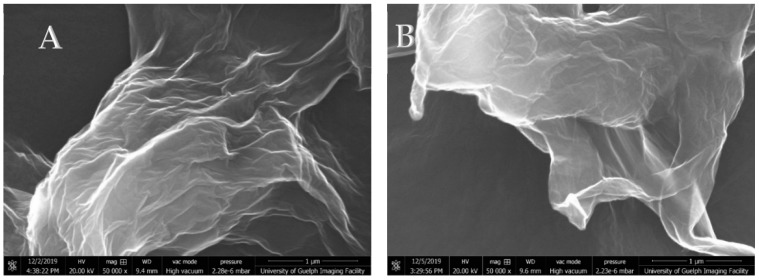
Scanning electron microscopy (SEM) images of (**A**) graphene oxide; (**B**) fluorinated graphene oxide (F-GO).

Scanning electron microscopy (SEM)was employed to characterize the surface morphologies and roughness. Figure 1A,B display the representative SEM images of the GO and F-GO recorded at a high magnification (50,000X). Both images showed the typical two-dimensional (2D) graphene oxide morphologies, which consist of crumbled and folded textures, showing irregular edges and rough surfaces. The synthesized TrGO, B-rGO and N-rGO exhibited a similar morphology to the GO and F-GO.

**Figure 2:**
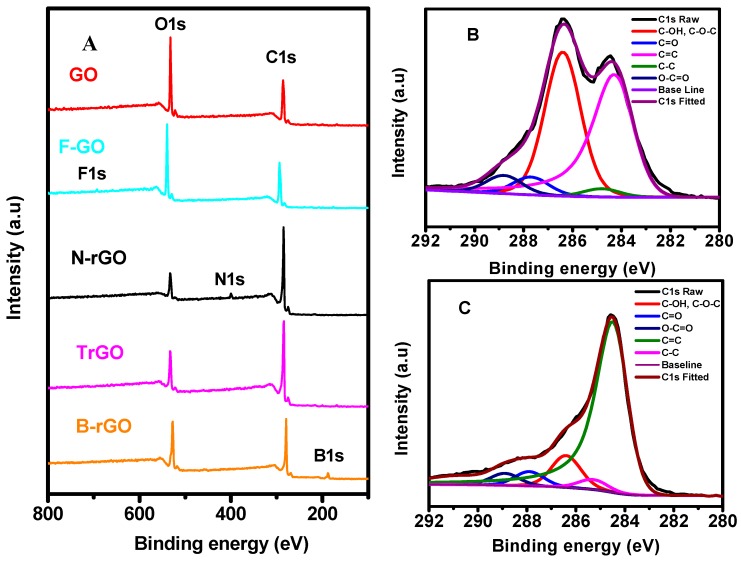
X-ray photoelectron spectroscopy (XPS) analysis of: (**A**) Survey spectra of graphene oxide (GO), fluorine-doped graphene oxide (F-GO), nitrogen-doped partially reduced graphene oxide (N-rGO), thermally reduced graphene oxide (TrGO), and boron-doped partially reduced graphene oxide (B-rGO). High-resolution C1s spectra with the deconvoluted peaks for (**B**) GO and (**C**) TrGO.

X-ray photoelectron spectroscopic analysis was performed on the GO and all the modified GO-samples to determine their composition and functional group species toward rationalizing their catalytic performance on the electrochemical oxidation of naproxen. Subsequently, the obtained XPS spectra were deconvoluted using Lorentzian and Gaussian functions [14,31]. Figure 2A displays the survey spectra of the GO, TrGO, F-GO, B-rGO and N-rGO, where strong O1s and C1s peaks appeared. The binding energy of C1s was increased in the following order: B-rGO (279.1 eV) < TrGO (284.3 eV) < N-rGO (285.1 eV) < GO (286.2 eV) < F-GO (293.7 eV). The binding energy of O1s was changed in the following order: B-rGO (527.1 eV) < GO (532.2 eV) < TrGO (532.3 eV) < N-rGO (533.1 eV) < F-GO (539.7 eV). Investigations had shown that the incorporation of boron into carbon materials was responsible for the lower binding energy peak for C 1s region, which could appear as broadening of the main peak or appear as a separate feature from the survey scan [32]. In contrast, doping with more electronegative atoms (e.g., N, F) into graphene oxide would decrease the electron density around carbon atoms, shifting the binding energy peak positively [32,33]. The observed order can be further explained by the amount of oxygen functional groups present. It has been reported that sp2 carbon and sp3 carbon binding energy of graphene oxide from the deconvoluted C1s peak appear in the range of 284.6 eV ± 0.3 eV, whereas carbon-bound oxygen functional groups appear at higher energy due to oxygen’s electronegativity [14,15]. In addition, the B1s peak of B-rGO was observed at 193.3 eV; the N1s peak of the N-rGO was seen at 400.1 eV, while a small F1s peak of the F-GO appeared at 692.7 eV. The presence of the F, N and B in the modified GO-based nanomaterials was further confirmed by the high-resolution XPS spectra of F1s, N1s and B1s as displayed in the supplementary material (Figure S1). The compositions of the GO and the modified-GO nanomaterials were calculated based on the survey spectra and listed in Table 1. The oxygen atomic percentage was decreased in the following order: GO (30.89%) > F-GO (29.97%) > B-rGO (21.63%) > TrGO (15.39%) > N-rGO (10.12%). The carbon atomic percentage was also changed due to the reduction and doping. The high-resolution C1s spectra of GO and TrGO are displayed in Figure 2B,C, respectively. The deconvoluted C1s peaks of GO and TrGO for each functional group contribution were C=C (284.3 eV), C–C (284.8 eV), C–O (286.4 eV), C=O (287.7 eV), O–C=O (288.8 eV) (Figure 2B) and C=C (284.5 eV), C–C (285.3 eV), C–O (286.4 eV), C=O (287.9 eV), and O–C=O (288.9 eV). The aforementioned XPS results were consistent with the deconvoluted values of GO reported in literature [14,32]. The high-resolution C1s spectra and the associated deconvoluted peaks of N-rGO, B-rGO and F-GO are presented in Figures S2–S4, respectively. As expected, the reduction of GO removed most epoxy and hydroxyl groups, while retaining most of the carboxyl and carbonyl groups [16]. As a consequence, numerous sp2 carbons were regenerated upon the cleavage of epoxy and hydroxyl functional groups, which was strongly evident when compared to the deconvoluted C1s peaks of GO (Figure 2B) and rGO (Figure 2C). The area under the sp2 carbon functional group curves occupied a much a larger percentage, whereas the epoxy/hydroxyl groups were significantly decreased. In contrast, the other functional groups were reduced by a much lower degree. The XPS spectra for the other modified graphene electrodes demonstrated the same trend based on the remaining quantity of functional oxygen groups following reduction.

### 3.2. Electrochemical Characterization of the Fabricated Various GO-Based Electrodes 

**Figure 3:**
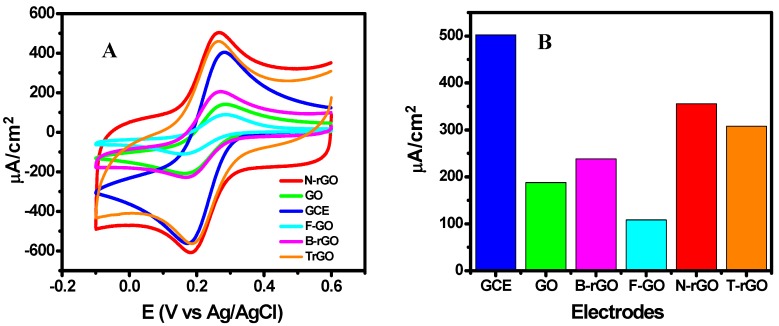
(**A**) Cyclic voltammograms for different electrodes recorded in a KNO_3_-ferricyanide media (5 mM K_3_[Fe(CN)_6_] in 0.2 M KNO_3_ at a scan rate of 50 mV/s; (**B**) Bar graph of the peak current densities of the different electrodes.

The modified electrodes (F-GO/GCE, B-rGO/GCE, N-rGO/GCE, GO/GCE, and TrGO/GCE) and pristine GCE were examined in a KNO_3_-ferricyanide medium (5 mM K_3_[Fe(CN)_6_] in 0.2 M KNO_3_) to compare their electrochemical performance. As seen in Figure 3A, the redox peak separation of the F-GO/GCE, GO/GCE, B-rGO/GCE, N-rGO/GCE, TrGO/GCE, and GCE was measured from the CV curves to be 129, 121, 104, 86, 100, and 99 mV, respectively. Figure 3B presents a comparison of the anodic peak current of these electrodes. The largest peak separations were demonstrated by the F-GO/GCE and GO/GCE, indicating a poor electron transfer efficiency. It is recognized that the doping of GO with fluorine is quite different from the doping with other heteroatoms, as boron as fluorine cannot substitute carbon atoms. Consequently, the incorporation of fluorine atoms generally disrupted additional sp2 carbon pi systems [34,35]. Similarly, graphene oxide typically demonstrates poor electron transfer efficiencies due to excess oxygen groups on graphene sheets, as they tend to disrupt sp2 carbon pi systems. This caused the low conductivities of F-GO and GO, which resulted in a low current density for both electrodes as shown in Figure 3B. Based on the XPS results listed in Table 1, the observed trend may be reiterated as follows: as fewer oxygen atoms are present due to reduction on the graphene sheet, the electron transfer efficiencies and current densities increases, in that more sp2 carbon structures are regenerated. It is therefore logical to verify whether this trend might apply to the catalytic oxidation of naproxen through the use of these electrodes.

### 3.3. Electrochemical Behaviors of Naproxen at the Modified Electrodes

**Scheme 1:**
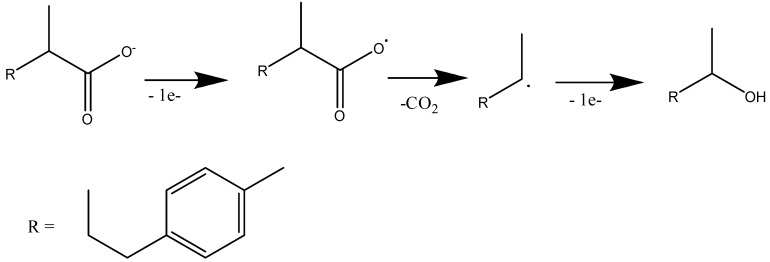
Electrochemical oxidation of naproxen.

**Figure 4:**
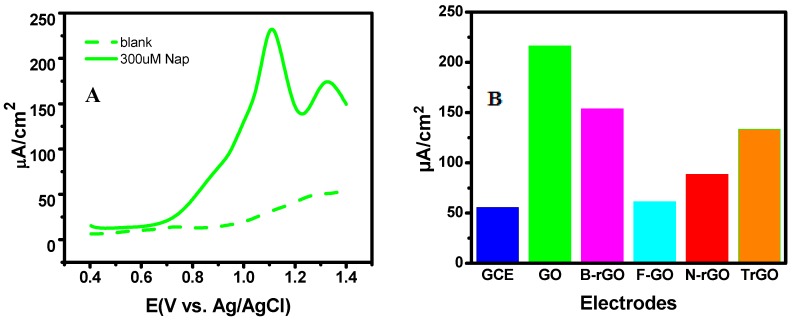
(**A**) Differential pulse voltammetry (DPV) curve for the detection of naproxen at 300 µM using GO/GCE. The dashed line is the blank measurement in the absence of naproxen. The solid line is the detection signal in the presence of 300 µM naproxen. (**B**) Bar graph for the electrochemical performance of the examined electrodes.

The CV curves recorded in a 0.1M PBS buffer (pH 7.2) in the absence (dashed line) and in the presence of 300 µM naproxen are presented in the supplementary materials (Figure S5). The first shoulder peak at ~0.86 V could be attributed to the electrochemical oxidation of naproxen, where one-electron oxidation process proceeds with the formation of naproxen cation radicals. The second shoulder peak at ~1.17 V might be due to the further oxidation of the cation radicals to form the ketone (2-acetyl-6-methoxynaphthalene) [10]. However, the observed CV peaks were not so clear. Thus, differential pulse voltammetry (DPV) was carried out in order to achieve a higher detection level. The DPV technique is based on the premise that the decay rate of capacitive current is much faster than Faradaic current, thus limiting the background current [36,37]. Figure 4A illustrates the detection of naproxen at 300 µM in 0.1M PBS (pH 7.2) using DPV. Two well-defined peaks were observed with the first peak being the major product following its decarboxylation [10,38], which is illustrated in Scheme 1.

Figure 4B compares the electrochemical performance of the different electrodes towards the oxidation of 300 µM naproxen. The GO/GCE electrode exhibited the highest performance, with a peak current density of 216.41 µA/cm^2^ at 1.13 V. In contrast, the GO/GCE generated the second lowest current density (188.00 µA/cm^2^) and the second largest peak separation (121.0 mV) during the ferricyanide test (Figure 3a). Similarly, F-GO/GCE demonstrated the lowest performance with a peak current density of 108.39 µA/cm^2^ and the largest peak separation of 129.00 mV in the ferricyanide test; however, the electrode exhibited a better performance over GCE for the detection of naproxen. The GCE generated the lowest signal at 55.52 µA/cm^2^ for the detection of naproxen although it showed the highest peak current density of 502.43 µA/cm^2^ in the ferricyanide test. The aforementioned results revealed that the oxygen content and dopants played critical roles in their electrochemical performance, showing that the percentage of oxygen (the epoxy and hydroxyl groups, in particular) was the main contributor to the significant differences in the catalytic oxidation performance of the modified GO electrodes. For instance, the GO/GCE possessed 30.89% of the oxygen content compared to 15.39% for the TrGO. The rationale behind the performance of these electrodes may be that as the oxygen content of graphene becomes higher, the catalytic oxidation of naproxen becomes stronger. The only outlier was the F-GO/GCE, as it accounted for only 28.33% of the GO detection signal. However, it is known that fluorine doping completely disrupts the sp2 carbon ring structure, thus further decreasing the conductivity of GO [13]. In comparison, nitrogen or boron-doped graphene oxide typically possessed heteroatoms that replaced the carbon atoms, and became incorporated within the ring structures [39]. According to the frontier molecular orbital theory, the lowest unoccupied molecular orbital (LUMO) of a fluorine-carbon bond would be higher than carbon-carbon or carbon-oxygen molecular orbitals, which would cause a decrease in the oxidation tendencies of organic molecules for the fluorinated graphene oxide. Furthermore, Park et al. reported that due to the significant differences in electronegativity between carbon and fluorine, the electrons from the valence band are transferred to the LUMO due to the high electronegativity of fluorine [29]. Due to this occupation by additional electrons polarizing LUMO, the oxidizing ability of the F-GO would decrease, which further explains its low electrochemical performance [40]. This characteristic is also supported by the electrochemical sensing of heavy metal ions using F-GO, in that the metal ions were first reduced at the F-GO surface and then stripped off from the surface [41].

### 3.4. Electrode Fouling 

**Figure 5:**
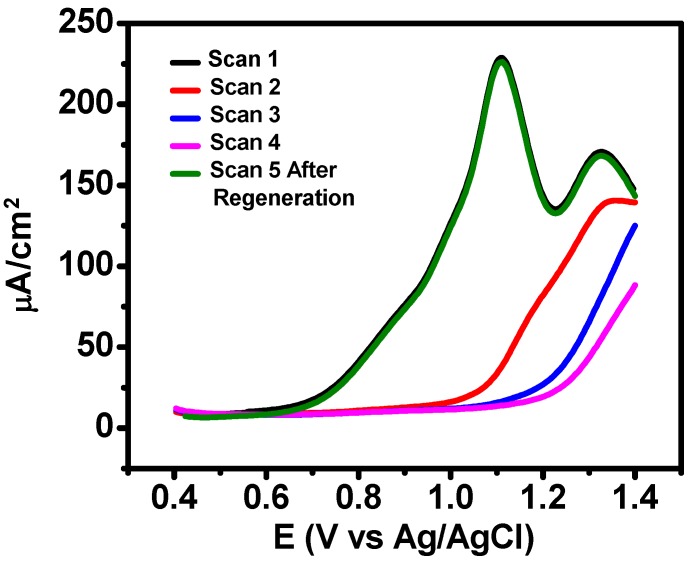
Differential Pulse Voltammetry (DPV) responses of the GO on Glassy Carbon Electrode(GCE) recorded in 0.1M PBS (pH 7.2) containing 300 µM naproxen.

The robust adsorbing behavior of naproxen is primarily facilitated by the binding interactions between the graphene sp2 carbon rings and carboxylic acid groups of naproxen [37]. Previous naproxen studies have not revealed a workable strategy toward a regenerable electrode for multiple detection events without a significant decrease in the signal current [33,37,38]. Certainly, the size of the naproxen molecule, and the fact that it contains hydrophobic (non-polar) benzene rings and multiple polar functional groups, predispose it to very easily foul electrodes [37]. Figure 5 illustrates this strong fouling effect following multiple scans with DPV, as the oxidized products adsorbed onto the electrode, resulting in the dramatic decrease of the current density of the electrochemical oxidation of naproxen during the 2nd, 3rd and 4th cycle. Most antifouling strategies involve a protective layer that may prevent fouling agents from reaching the electrode surface [42]. However, in our case, where the fouling agent is the analyte itself, this strategy is not viable. Electrochemical activation and surface modification are two strategies that may resolve this issue. Here, we designed an electrochemical activation strategy, which employed short pulses with high anodic potential for the removal of adsorbed species, while preventing oxygen evolution which might peel off the electrode coating. Specifically, this activation method adopted a multi-potential step approach with the following parameters: (i) initial potential at 0.0 V for 5 s; (ii) stepped to 2.8 V for 50 ms; (iii) stepped down to 0.0 V for 5 s; and (iv) repeated over 12 cycles. As shown in Figure 5, after the regeneration process, the 5th DPV curve was almost identical to the first scan, showing that the activation strategy effectively overcame the fouling issues during the electrochemical detection of naproxen.

### 3.5. Electrochemical Sensing of Naproxen

**Figure 6:**
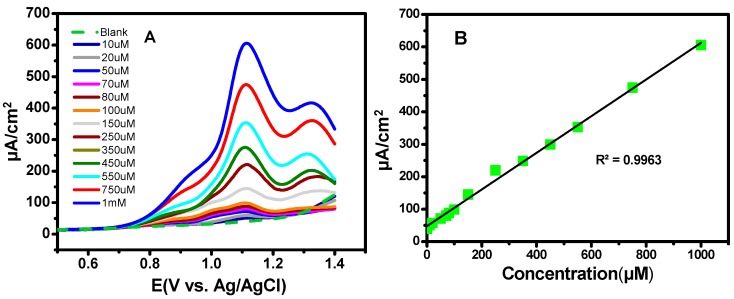
(**A)** DPV responses at different naproxen concentrations for the GO/GCE using DPV. (**B**) Calibration curve exhibits a linear response in current density as the concentration increased from 10 µM to 1 mM.

Figure 6A displays a series of DPV curves of the GO/GCE in a 10.0 ml of 0.1M PBS buffer (pH 7.2) with different naproxen concentrations, showing the current density was increased with the increase of the concentration. The associated calibration plot from the concentrations, ranging from 10 µM to 1 mM, is presented in Figure 6B, with the R^2^ value of 0.9963, which signified a very strong linear relationship. The sensitivity of the sensor was obtained from the slope of the regression line to be 0.60 µAµM^−1^cm^−2^. The limit of detection (LOD) was calculated to be 1.94 µM, which was acquired using the formula LOD = 3 σ/s. The limit of quantification (LOQ) was calculated to be 6.47 µM, which was obtained using the formula LOQ = 10 σ/s, where σ represents the standard deviation of the five blank measurements, and s denotes the slope from the calibration curve.

### 3.6. Interference Studies and Real Sample Analysis

**Figure 7:**
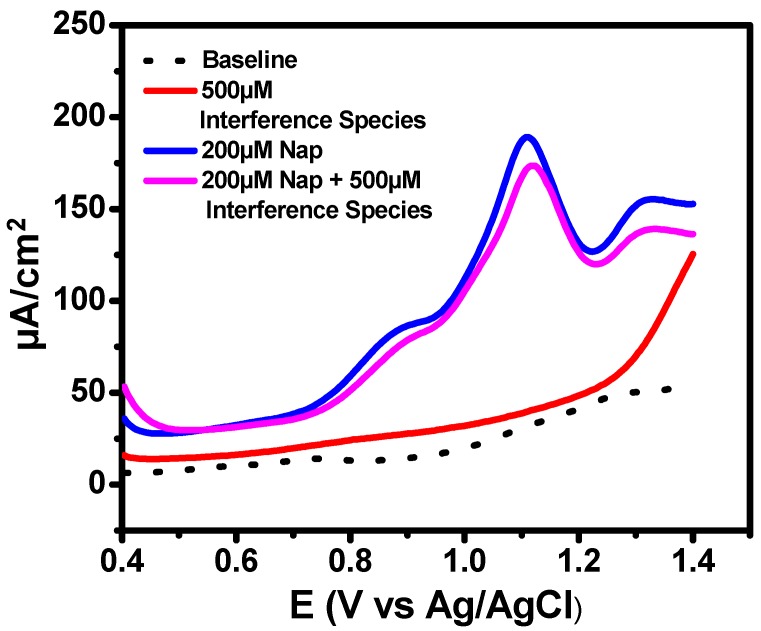
DPV responses showing the responses of 200 M naproxen in the presence and absence of 500 µM chloride ions, nitrate ions, glutamic acid, glycin, citric acid, sulfate ions, ascorbic acid, and D-glucose interference species.

The selectivity of the developed GO/GCE sensor was also investigated; Figure 7 presents the DPV response to 200 µM naproxen in the presence of chloride ions, nitrate ions, glutamic acid, glycin, citric acid, sulfate ions, ascorbic acid, and D-glucose (500 µM each). No notable response to the interference species was observed. The GO/GCE exhibited 91% of the oxidation signal compared to pure naproxen, showing that the sensor had excellent selectivity towards the detection of naproxen. 

**Figure 8:**
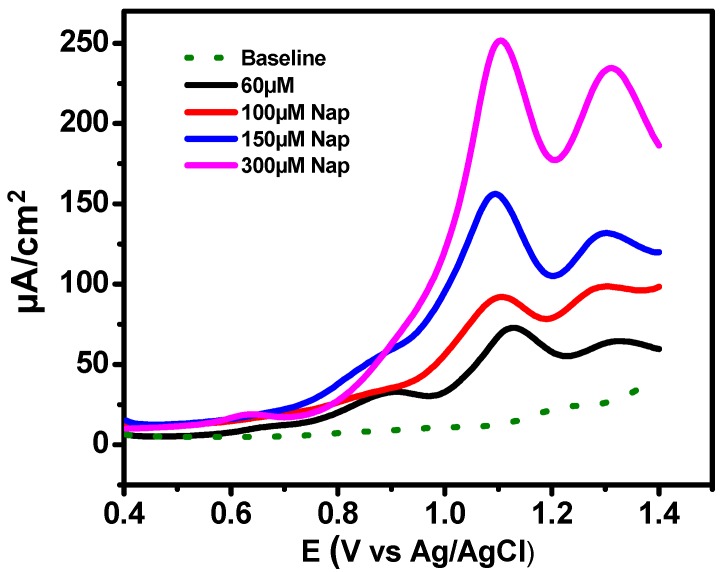
DPV responses showing the detection of 30 µM, 100 µM, 150 µM, and 300 µM naproxen.

The performance of the GO/GCE sensor was further tested using a Life Brand Naproxen tablet (220 mg) as shown in Figure 8. The tablet was dissolved in 0.1 M NaOH as the preparation of the naproxen stock solution. There were no apparent interference species from the excipients in the tablets. The peak current density measured from the DPV curves (Figure 8) was fitted to the calibration plot (Figure 6B) for comparison. Multiple measurements at the same concentration were conducted, and a mean recovery of 96.9% with a relative standard deviation of 2.5% was obtained. These results verified a precise and accurate electrochemical quantification of the sensor.

## 4. Conclusions

In summary, five different GO-based nanomaterials including GO, TrGO, B-rGO, N-rGO and F-GO were synthesized and systemically studied. GO/GCE exhibited the strongest activity toward the electrochemical oxidation of naproxen compared to GCE and other modified GO electrodes. This was primarily due to an enhanced catalytic activity facilitated by the oxygen functional groups of GO, particularly the epoxy and hydroxyl groups, which was confirmed by the XPS analysis. The sensitive quantification of naproxen was successfully achieved over a wide linear concentration range from 10 µM to 1 mM by DPV. Naproxen had very strong propensity for fouling electrode surfaces, resulting in a substantial decrease of the current. A facile electrochemical activation strategy based on potential pulses was developed, which successfully overcame the critical fouling problem. The GO/GCE sensor developed in this study exhibited high sensitivity and selectivity over a wide range of concentrations of naproxen. Off-the-shelf naproxen tablets were successfully detected using the developed sensor, which demonstrated strong anti-interference capabilities. Future studies should be conducted to elucidate the specific naproxen catalytic kinetics with oxygenated functional groups, as well as heteroatom dopant levels, catalytic oxidation and antifouling properties toward naproxen.

## Figures and Tables

**Table 1:** Summary of atomic percentages of the synthesized graphene oxide based nanomaterials.

GO-Based Nanomaterials	Carbon Atomic %	Oxygen Atomic %	Heteroatom Atomic %
**GO**	69.11	30.89	
**TrGO**	84.61	15.39	
**B-rGO**	64.92	23.15	11.93
**F-GO**	69.36	29.97	0.67
**N-rGO**	86.91	10.12	2.97

Graphene Oxide (GO), Thermally Reduced Graphene Oxide (TrGO), Boron-doped partially Reduced Graphene Oxide (B-rGO), Fluorine-doped partially Reduced Graphene Oxide (F-rGO), Nitrogen-doped partially Reduced Graphene Oxide (N-rGO).

## Supplementary Materials

The following are available online at https://www.mdpi.com/1424-8220/20/5/1252/s1. Figure S1. High-resolution XPS spectra of: (A) F1s peak of fluorinated graphene oxide at 692.675; (B) N1s peak of nitrogen doped reduced graphene oxide at 400.137 eV; and (C) B1s peak of boron doped reduced graphene oxide at 197.455 eV. Figure S2: High-resolution C1s spectra with deconvoluted peaks for N-rGO. Figure S3: High-resolution C1s spectra with deconvoluted peaks for B-rGO. Figure S4: High-resolution C1s spectra with deconvoluted peaks for F-GO. Figure S5: CV curves of GO/GCE recorded at a scan rate of 50 mV/s in 0.1M PBS (pH 7.2) buffer in the absence of (dashed line) and in the presence of 300 µM Naproxen (solid line). Figure S6. DPV curves of the various electrodes recorded in a solution 0.1M PBS (pH 7.2) containing 300 µM of Naproxen.

## References

[1] Angiolillo D.J., Weisman S.M. (2017). Clinical pharmacology and cardiovascular safety of naproxen. Am. J. Cardiovasc. Drugs.

[2] Jain P.S. (2019). A concise review on analytical profile of naproxen. IP Int. J. Compr. Adv. Pharmacol..

[3] Janssens H.J.E.M., Janssen M., van de Lisdonk E.H., van Riel P.L.C.M., van Weel C. (2008). Use of oral prednisolone or naproxen for the treatment of gout arthritis: A double-blind, randomised equivalence trial. Lancet.

[4] Al-Abri S.A., Anderson I.B., Pedram F., Colby J.M., Olson K.R. (2015). Massive naproxen overdose with serial serum levels. J. Med Toxicol..

[5] Isidori M., Lavorgna M., Nardelli A., Parrella A., Previtera L., Rubino M. (2005). Ecotoxicity of naproxen and its phototransformation products. Sci. Total Environ..

[6] Brozinski J.M., Lahti M., Meierjohann A., Oikari A., Kronberg L. (2013). The anti-inflammatory drugs diclofenac, naproxen and ibuprofen are found in the bile of wild fish caught downstream of a wastewater treatment plant. Environ. Sci. Technol..

[7] Ostojic J., Herenda S., Besic Z., Milos M., Galic B. (2017). Advantages of an electrochemical method compared to the spectrophotometric kinetic study of peroxidase inhibition by boroxine derivative. Molecules.

[8] Afkhami A., Kafrashi F., Ahmadi M., Madrakian T. (2015). A new chiral electrochemical sensor for the enantioselective recognition of naproxen enantiomers using l-cysteine self-assembled over gold nanoparticles on a gold electrode. RSC Adv..

[9] Baj-Rossi C., Rezzonico Jost T., Cavallini A., Grassi F., De Micheli G., Carrara S. (2014). Continuous monitoring of Naproxen by a cytochrome P450-based electrochemical sensor. Biosens. Bioelectron..

[10] Hendawy H.A.M., Salem W.M., Abd-Elmonem M.S., Khaled E. (2019). Nanomaterial-based carbon paste electrodes for voltammetric determination of naproxen in presence of its degradation products. J. Anal. Methods Chem..

[11] Zhu C., Yang G., Li H., Du D., Lin Y. (2015). Electrochemical sensors and biosensors based on nanomaterials and nanostructures. Anal. Chem..

[12] Coroş M., Pogăcean F., Măgeruşan L., Socaci C., Pruneanu S. (2019). A brief overview on synthesis and applications of graphene and graphene-based nanomaterials. Front. Mater. Sci..

[13] Rao S., Upadhyay J., Polychronopoulou K., Umer R., Das R. (2018). Reduced graphene oxide: Effect of reduction on electrical conductivity. J. Compos. Sci..

[14] Zhang Z., Schniepp H.C., Adamson D.H. (2019). Characterization of graphene oxide: Variations in reported approaches. Carbon.

[15] Priya Swetha P.D., Manisha H., Sudhakaraprasad K. (2018). Graphene and graphene-based materials in biomedical science. Part. Part. Syst. Charact..

[16] Wang Z., Pu Y., Wang D., Wang J.-X., Chen J.-F. (2018). Recent advances on metal-free graphene-based catalysts for the production of industrial chemicals. Front. Chem. Sci. Eng..

[17] Lee M., Yang S., Kim K.-j., Kim S., Lee H. (2014). Comparison of the catalytic oxidation reaction on graphene oxide and reduced graphene oxide. J. Phys. Chem. C.

[18] Kong X.K., Chen C.L., Chen Q.W. (2014). Doped graphene for metal-free catalysis. Chem. Soc. Rev..

[19] Tian W.C., Li W.H., Yu W.B., Liu X.H. (2017). A review on lattice defects in graphene: Types, generation, effects and regulation. Micromachines.

[20] Feicht P., Eigler S. (2018). Defects in graphene oxide as structural motifs. ChemNanoMat.

[21] Manikandan V.S., Adhikari B., Chen A. (2018). Nanomaterial based electrochemical sensors for the safety and quality control of food and beverages. Analyst.

[22] Adhikari B.-R., Govindhan M., Chen A. (2015). Sensitive detection of acetaminophen with graphene-based electrochemical sensor. Electrochim. Acta.

[23] Liu Z., Puumala E., Chen A. (2019). Sensitive electrochemical detection of Hg(II) via a FeOOH modified nanoporous gold microelectrode. Sens. Actuators B Chem..

[24] Li D., Wang T., Li Z., Xu X., Wang C., Duan Y. (2019). Application of graphene-based materials for detection of nitrate and nitrite in water-a review. Sensors.

[25] Huang H., Su S., Wu N., Wan H., Wan S., Bi H., Sun L. (2019). Graphene-based sensors for human health monitoring. Front. Chem..

[26] Luo L., Peng T., Yuan M., Sun H., Dai S., Wang L. (2018). Preparation of graphite oxide containing different oxygen-containing functional groups and the study of ammonia gas sensitivity. Sensors.

[27] Chen A., Chatterjee S. (2013). Nanomaterials based electrochemical sensors for biomedical applications. Chem. Soc. Rev..

[28] Qureshi T.S., Panesar D.K., Sidhureddy B., Chen A., Wood P.C. (2019). Nano-cement composite with graphene oxide produced from epigenetic graphite deposit. Compos. Part B Eng..

[29] Thiruppathi A.R., Sidhureddy B., Keeler W., Chen A. (2017). Facile one-pot synthesis of fluorinated graphene oxide for electrochemical sensing of heavy metal ions. Electrochem. Commun..

[30] Guo H.-L., Su P., Kang X., Ning S.-K. (2013). Synthesis and characterization of nitrogen-doped graphene hydrogels by hydrothermal route with urea as reducing-doping agents. J. Mater. Chem. A.

[31] Iakovlev V.Y., Sklyueva Y.A., Fedorov F.S., Rupasov D.P., Kondrashov V.A., Grebenko A.K., Mikheev K.G., Gilmutdinov F.Z., Anisimov A.S., Mikheev G.M. (2018). Improvement of optoelectronic properties of single-walled carbon nanotube films by laser treatment. Diam. Relat. Mater..

[32] Susi T., Pichler T., Ayala P. (2015). X-ray photoelectron spectroscopy of graphitic carbon nanomaterials doped with heteroatoms. Beilstein J. Nanotechnol..

[33] Zhan C., Zhang Y., Cummings P.T., Jiang D.E. (2016). Enhancing graphene capacitance by nitrogen: Effects of doping configuration and concentration. Phys. Chem. Chem. Phys..

[34] Huang X., Hu N., Zhang L., Wei L., Wei H., Zhang Y. (2013). The NH_3_ sensing properties of gas sensors based on aniline reduced graphene oxide. Synth. Met..

[35] Zhao F.G., Zhao G., Liu X.H., Ge C.W., Wang J.T., Li B.L., Wang Q.G., Li W.S., Chen Q.Y. (2014). Fluorinated graphene: Facile solution preparation and tailorable properties by fluorine-content tuning. J. Mater. Chem. A.

[36] Chen A., Shah B. (2013). Electrochemical sensing and biosensing based on square wave voltammetry. Anal. Methods.

[37] Sandford C., Edwards M.A., Klunder K.J., Hickey D.P., Li M., Barman K., Sigman M.S., White H.S., Minteer S.D. (2019). A synthetic chemist’s guide to electroanalytical tools for studying reaction mechanisms. Chem. Sci..

[38] Suryanarayanan V., Zhang Y., Yoshihara S., Shirakashi T. (2005). Voltammetric assay of naproxen in pharmaceutical formulations using boron-doped diamond electrode. Electroanalysis.

[39] Wang X., Sun G., Routh P., Kim D.H., Huang W., Chen P. (2014). Heteroatom-doped graphene materials: Syntheses, properties and applications. Chem. Soc. Rev..

[40] Park M.-S., Kim K.H., Kim M.-J., Lee Y.-S. (2016). NH 3 gas sensing properties of a gas sensor based on fluorinated graphene oxide. Colloids Surf. A Physicochem. Eng. Asp..

[41] Lach J., Szymonik A. (2019). Adsorption of naproxen sodium from aqueous solutions on commercial activated carbons. J. Ecol. Eng..

[42] Hanssen B.L., Siraj S., Wong D.K.Y. (2016). Recent strategies to minimise fouling in electrochemical detection systems. Rev. Anal. Chem..

