# Supplementary material for: Graphene-Oxide-Based Electrochemical Sensors for the Sensitive Detection of Pharmaceutical Drug Naproxen
Source: Sensors (Basel). 2020 Feb 25;20(5):1252. doi: 10.3390/s20051252 (PMC7085571; doi:10.3390/s20051252)
Supplement: Supplementary file 1 [file sensors-20-01252-s001.pdf]

Supplementary Materials

Graphene-Oxide-Based Electrochemical Sensors for the Sensitive Detection of Pharmaceutical Drug Naproxen

Lanting Qian, Antony Raj Thiruppathi, Reem Elmahdy, Joshua van der Zalm and Aicheng Chen*

Electrochemical Technology Centre, Department of Chemistry, University of Guelph, 50 Stone Road East, Guelph, Ontario N1G 2W1, Canada; qianl@uoguelph.ca; athirupp@uoguelph.ca; relmahdy@uoguelph.ca; vanderzj@uoguelph.ca

* Correspondence: aicheng@uoguelph.ca ;Tel.: 1-519-8244120 Ext. 54764; Fax: 1-519-7661499;

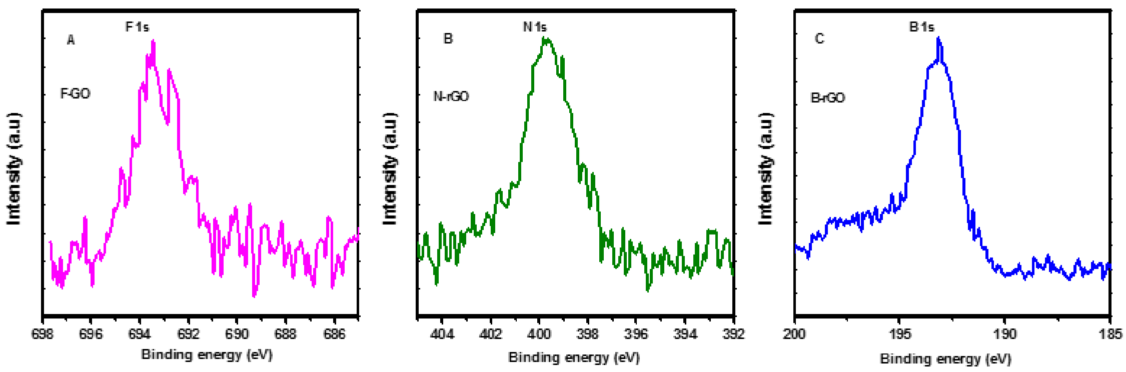

Figure S1. High-resolution XPS spectra of: (**A**) F1s peak of fluorinated graphene oxide at 692.675; (**B**) N1s peak of nitrogen doped reduced graphene oxide at 400.137 eV; and (**C**) B1s peak of boron doped reduced graphene oxide at 197.455 eV.

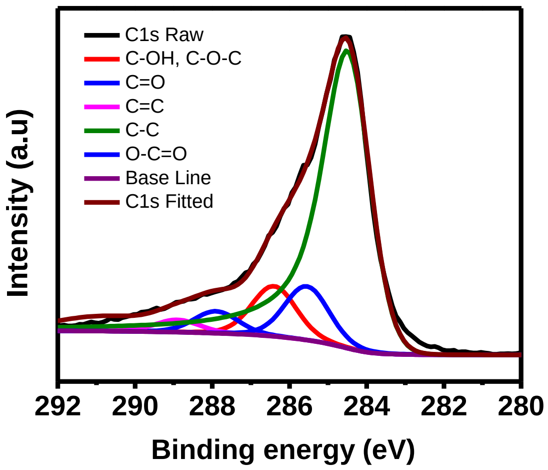

Figure S2. High-resolution C1s spectrum with the deconvoluted peaks for N-rGO.

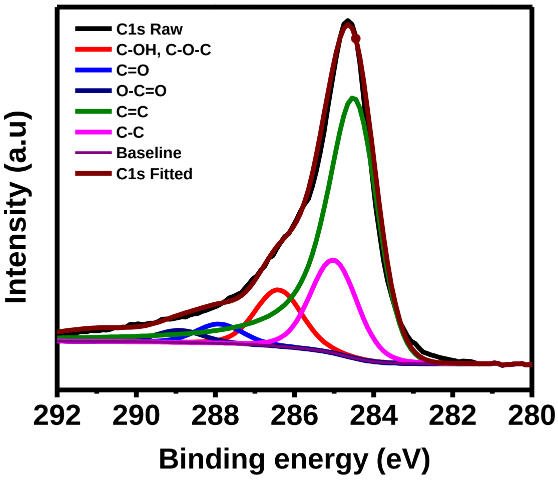

Figure S3. High-resolution C1s spectrum with the deconvoluted peaks for B-rGO.

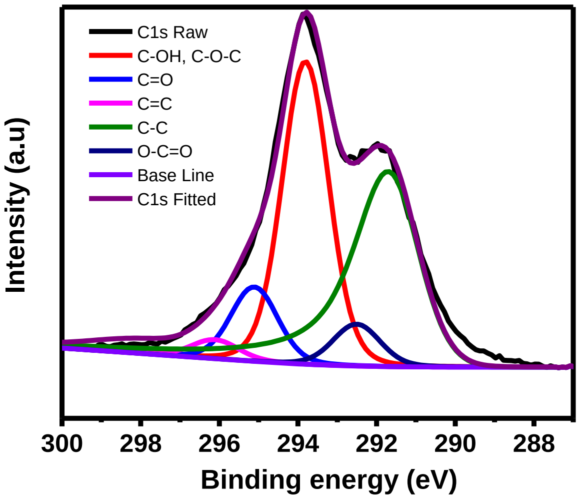

Figure S4. High-resolution C1s spectrum with the deconvoluted peaks for F-GO.

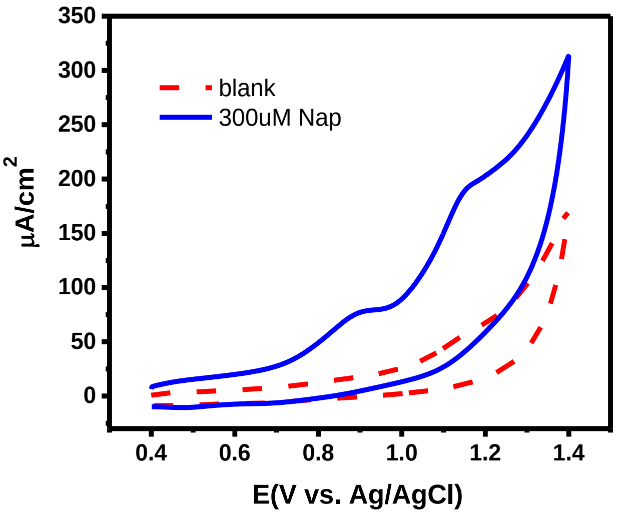

Figure S5. CV curves of GO/GCE recorded at a scan rate of 50 mV/s in 0.1M PBS (pH 7.2) buffer in the absence of (dashed line) and in the presence of 300 μM Naproxen (solid line).

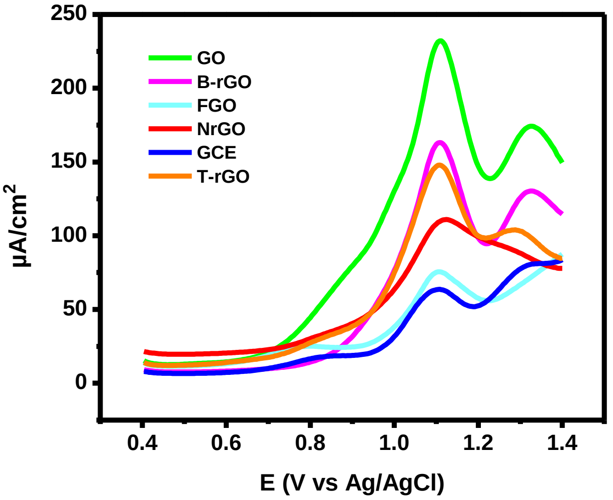

Figure S6. DPV curves of the various electrodes recorded in 0.1M PBS (pH 7.2) containing 300 μM.
